# Observations on the May 2019 Joffre Peak landslides, British Columbia

**DOI:** 10.1007/s10346-019-01332-2

**Published:** 2020-01-02

**Authors:** Pierre Friele, Tom H. Millard, Andrew Mitchell, Kate E. Allstadt, Brian Menounos, Marten Geertsema, John J. Clague

**Affiliations:** 1 Cordilleran Geoscience, PO Box 612, Squamish, BC V8B 0A5 Canada; 2 BC Ministry of Forests Lands Natural Resource Operations and Rural Development (BC FLNRORD), 2100 Labieux Rd #103, Nanaimo, BC V9T 6E9 Canada; 3 Department of Earth, Ocean and Atmospheric Sciences, University of British Columbia, 2207 Main Mall #2020, Vancouver, BC V6T 1Z4 Canada; 4 U.S. Geological Survey, Geologic Hazards Science Center, Box 25046, DFC, MS 966, Denver, CO 80225 USA; 5 Geography Program and Natural Resources and Environmental Studies Institute, University of Northern British Columbia, 3333 University Way, Prince George, BC V2N 4Z9 Canada; 6 BC Ministry of Forests Lands Natural Resource Operations and Rural Development, 2000 Ospika Blvd S, Prince George, BC V2N 4W5 Canada; 7 Department of Earth Sciences, Simon Fraser University, 8888 University Drive, Burnaby, BC V5A 1S6 Canada

**Keywords:** Landslide, Rock avalanche, Debris flow, Flood, Seismic analysis, Snowmelt, Permafrost, Travel angle, Joffre Peak, British Columbia

## Abstract

Two catastrophic landslides occurred in quick succession on 13 and 16 May 2019, from the north face of Joffre Peak, Cerise Creek, southern Coast Mountains, British Columbia. With headscarps at 2560 m and 2690 m elevation, both began as rock avalanches, rapidly transforming into debris flows along middle Cerise Creek, and finally into debris floods affecting the fan. Beyond the fan margin, a flood surge on Cayoosh Creek reached bankfull and attenuated rapidly downstream; only fine sediment reached Duffey Lake. The toe of the main debris flow deposit reached 4 km from the headscarp, with a travel angle of 0.28, while the debris flood phase reached the fan margin 5.9 km downstream, with a travel angle of 0.22. Photogrammetry indicates the source volume of each event is 2–3 Mm^3^, with combined volume of 5 Mm^3^. Lidar differencing, used to assess deposit volume, yielded a similar total result, although error in the depth estimate introduced large volume error masking the expected increase due to dilation and entrainment. The average velocity of the rock avalanche-debris flow phases, from seismic analysis, was ~ 25–30 m/s, and the velocity of the 16 May debris flood on the upper fan, from super-elevation and boulder sizes, was 5–10 m/s. The volume of debris deposited on the fan was ~ 10^4^ m^3^, 2 orders of magnitude less than the avalanche/debris flow phases. Progressive glacier retreat and permafrost degradation were likely the conditioning factors; precursor rockfall activity was noted at least ~6 months previous; thus, the mountain was primed to fail. The 13 May landslide was apparently triggered by rapid snowmelt, with debuttressing triggering the 16 May event.

## Introduction

**Fig. 1:**
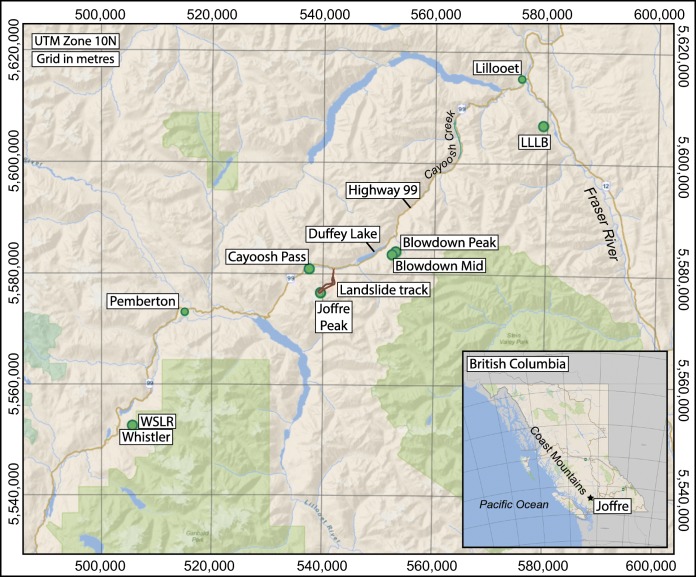
Location map showing Joffre Peak, the track of the landslides, place names mentioned in text, and locations of seismic and climate stations

On 13 and 16 May 2019, two large catastrophic rock avalanches occurred on the north slope of Joffre Peak, devastated the middle reach of Cerise Creek, and affected the alluvial fan. Joffre Peak (2720 m asl) is a towering peak within the Cerise Creek watershed, located in the Coast Mountains, southwest British Columbia (BC) (Fig. 1). Cerise Creek discharges into Cayoosh Creek, which in turn joins the Fraser River at the town of Lillooet.

**Fig. 2:**
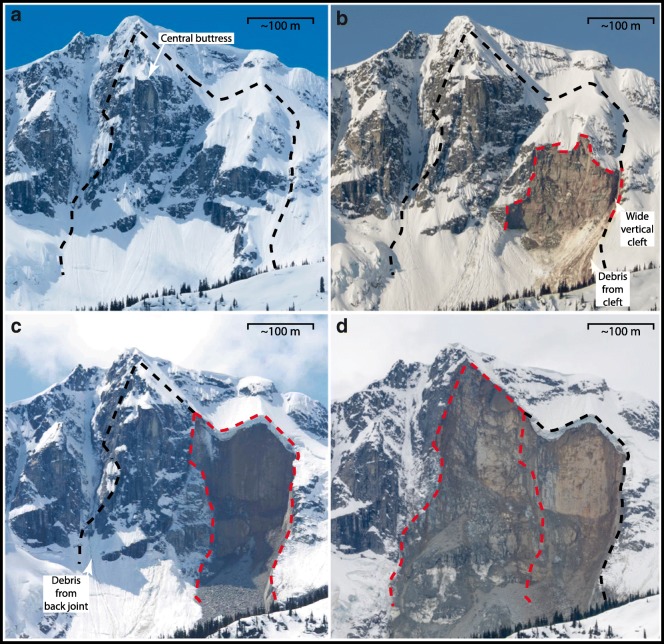
Photos of the north face of Joffre Peak from Highway 99. Black dashed line encompasses both scars; the red dash lines delineate sequential failures. Photographs taken on **a** 1 May 2017 and **b** 6 May 2019 by Ian Routley. The face partially collapsed sometime between these two dates. This collapse and the debris emanating from the wide vertical cleft on the fall-line left of it are indicative of precursor distress. **c** Photo of 13 May landslide taken on 14 May by Jay Mamay. **d** Photo of 16 May landslide taken on 19 May by Ian Routley. Photos are scaled the same for comparison of scars

**Fig. 3:**
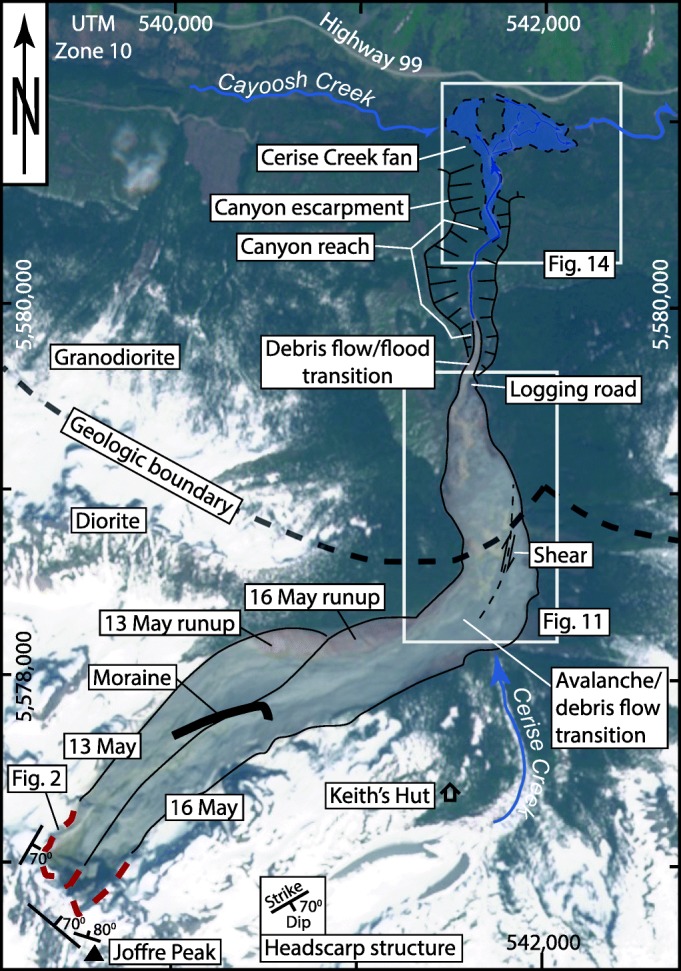
The 13 and 16 May landslide tracks, showing bedrock geology and structure, approximate locations of phase transitions, other features discussed in text, and locations of larger scale map areas (Figs. 11 and 14). The blue polygon on the fan is the affected area

The north face of Joffre Peak is visible from BC Highway 99 (Fig. 2), which connects the towns of Pemberton and Lillooet and is a scenic route through the Coast Mountains (Fig. 1). The road provides access to several popular backcountry recreation destinations, including Keith’s Hut, a mountaineering cabin in the Cerise Creek watershed (Fig. 3). Due to Joffre’s prominence and the high recreational use of the Cerise Creek watershed, citizen science observations, including photographs of precursor slope activity, initial observations of the two landslides, and post-event activity were posted on social media.

The Cerise Creek watershed and the Joffre Creek watershed on the south side of the peak have been designated conservation areas and fall under the jurisdiction of BC Parks. As such, BC Parks commissioned an overflight following the 13 May event, with an additional flight provided by the Ministry of FLNRORD following the 16 May event. The objectives of the flights were to assess the events and residual hazards and to make recommendations regarding public safety. During these flights, oblique photos were taken of the landslide’s headscarp and deposit.

This paper provides observations and preliminary analyses of the two landslides based on citizen science observations, structure from motion (sfm) photogrammetry models compiled from oblique photos taken during helicopter overview flights on 15 and 18 May 2019, Lidar surveys flown on 22 February 2018 and 26 May 2019, analysis of seismic signals from nearby seismometers, and reconnaissance field surveys on 27 May and 8 June 2019.

## Geology

Joffre Peak is composed of a middle Cretaceous diorite (Fig. 3; Roddick and Hutchinson 1973), part of the plutonic core of the Coast Mountains (Fig. 1). We mapped joint orientations in the landslide source areas from a photogrammetry model created from handheld photos acquired during post-event reconnaissance using the Compass plugin in CloudCompare (Thiele et al. 2017). The backscarps of both landslides corresponds to a foliation dipping 70° with a dip direction of 040°. The backscarp is deeply oxidized, indicating that chemical weathering likely contributed to progressive loss of strength. A joint set dipping 70° toward 120° bounds the lateral margin of the source area to the north. The rock mass is more highly fractured on the south side of the exposed scarp, and the south margin of the source area daylights along joint sets dipping between 70° and 85°, with dip directions between 355° to 035°. There are no mapped faults in the vicinity of the peak.

## Glacial history

The Coast Mountains were subject to alpine and ice sheet glaciation during the Quaternary Period. The landscape is one of deep U-shaped valleys, aretes, cirques, and other classic alpine glacial landforms. Till and colluvium mantle valley walls, and till, glaciolacustrine, glaciofluvial, and fluvial sediments underlie the valley floor forming a thick Quaternary fill. During the final Pleistocene glaciation, known locally as the Fraser Glaciation, British Columbia was mantled by the Cordilleran Ice Sheet, and only the highest peaks in the Coast Mountains, like the rugged summit of Joffre, stood above the surface of this ice sheet as nunataks. The ice sheet began to thin and retreat by 17 ka, and by 11.7 ka, glacier extent in the southern Coast Mountains was comparable to that of today (Menounos et al. 2017). Climate during the early Holocene was warmer and drier than today, but beginning about 7–8 ka, climate began to cool and precipitation increased (Mathewes and Heusser 1981). These changes in climate caused alpine glaciers to expand, with maximum ice cover achieved during the Little Ice Age advances of the eighteenth and nineteenth centuries (Koch et al. 2007; Menounos et al. 2009). Following these advances, glaciers in the southern Coast Mountains retreated during two intervals, the period 1920–1945 and after 1980 (deBeer and Sharp 2007; Schiefer et al. 2007; Koch et al. 2009). A recently abandoned Little Ice Age moraine lies at the foot of the north slope of Joffre Peak (Fig. 3). The moraine marks the maximum Holocene extent of a former glacier some 1500 m long that terminated at about 1600 m asl. By the time of the landslides, this glacier had retreated 700 m and comprised thin patches of ice above 1900 m asl.

## Landslide history

Large landslides are common throughout the Coast Mountains and include large historical events, for example, the 2010 Mount Meager debris avalanche (ca. 50 Mm^3^; Guthrie et al. 2012; Roberti et al. 2017a, b). While most prehistoric events have not been dated, we assume that the temporal pattern follows that described for other similar landscapes in the world, with increased landslide activity related to environmental drivers such as the Holocene paraglacial cycle (Blikra and Nemec 1998; Ballantyne et al. 2014; Hilger et al. 2018), a shift to cooler/wetter Neoglacial climate (Matthews et al. 2009), debuttressing due to thinning and retreat from Little Ice Age glacier limits (Evans and Clague 1994; Holm et al. 2004), recent human-induced climate warming (Huggel et al. 2011; Reid 2017), and earthquake activity (Mathews 1979). These pulses of activity are set against an otherwise stochastic background of landslide activity over time (Dadson and Church 2005), presumably controlled by rock mass weakening.

## Climate

**Fig. 4:**
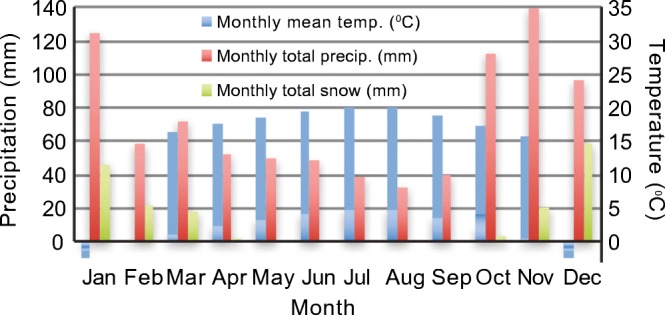
Temperature and precipitation at Pemberton (200 m asl; 1984-2006). Snow is water equivalent

Joffre Peak is located at the transition between a coastal maritime climate (e.g., the Village of Pemberton) and a continental climate (e.g., the Village of Lillooet) (Fig. 1). Summers are warm, winters cool, and the precipitation peak occurs in the fall/winter months when cyclonic storms are common. The nearest long-term climate station (https://climat.meteo.gc.ca/historical_data/) is at Pemberton (1984–present; 200 m asl; Fig. 1), where the mean annual temperature is 8 °C and mean annual precipitation is 870 mm with 170 mm falling as snow (Fig. 4). The online webtool ClimateBC (http://www.climatewna.com) suggests that Cayoosh Pass at 1200 m asl has a mean annual temp of 3.9 °C and mean annual precipitation of 950 mm, with 420 mm falling as snow. Corresponding estimates at 1800 m asl on the mid-slope of Joffre Peak are 1.2 °C and 1110 mm, with 705 mm of snow.

**Fig. 5:**
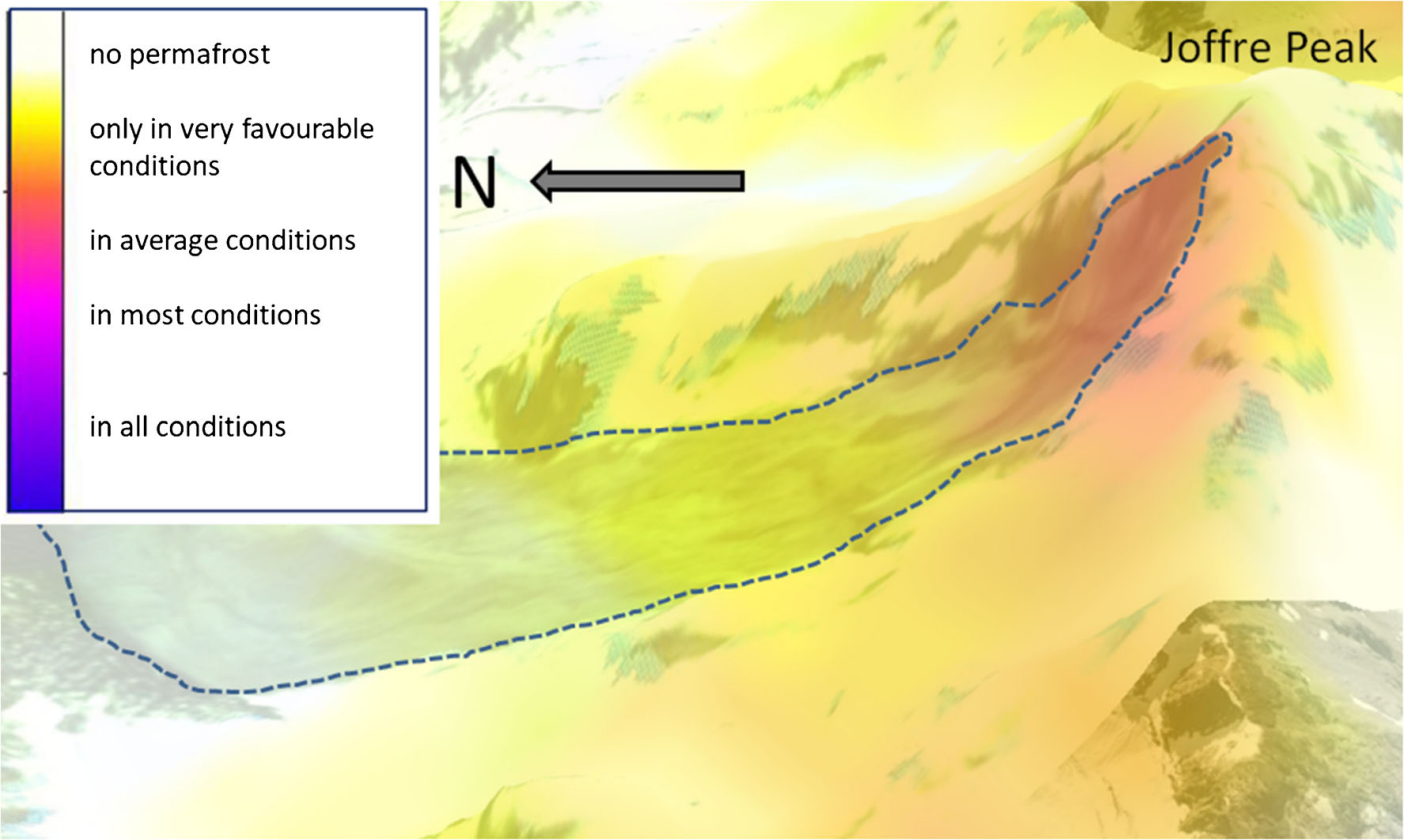
The landslides initiated from the north face of Joffre Peak in an area of expected mountain permafrost. This image is a blend of a DEM, 26 May 2019 Planet image (Planet Team 2019), and the provisional permafrost favourability map of Hasler and Geertsema (2013). The north face of Joffre Peak has strong permafrost favourability in contrast to its south face. Image is 3 km across in an E-W direction

ClimateBC data indicate that the 0 °C isotherm is at about 2050 m asl on the north side of Joffre Peak; however, this alone is not a direct indication of permafrost being present. The global permafrost of Gruber (2012 a, b) and the more detailed model of Hasler and Geertsema (2013), which combine ClimateBC data with elevation, aspect, and potentially incoming solar radiation, restrict permafrost favorability to the north face of Joffre Peak (Fig. 5), where the landslides initiated.

**Fig. 6:**
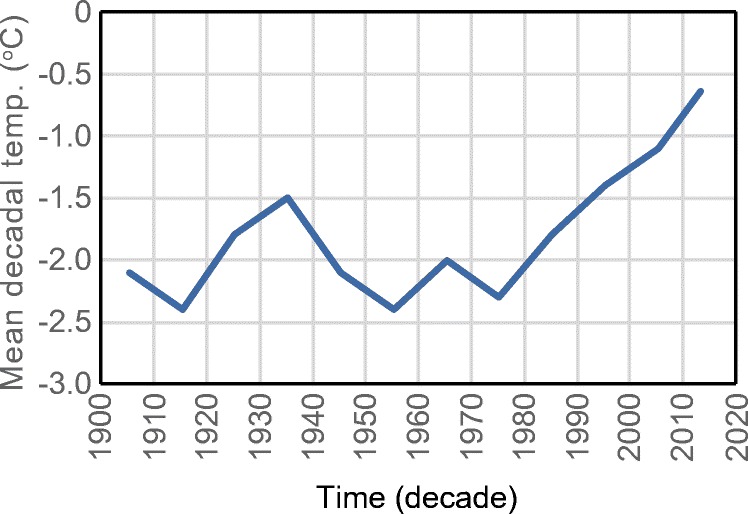
ClimateBC estimates of decadal average temperature, 1900–1910 to 2010–2020, north face of Joffre Peak at 2400 m asl. The last point is the average of 2011–2018. Note the positive trend starting after 1970–1980. This trend suggests permafrost degradation; with negative temperatures approaching zero, slope stability may have been vulnerable to rapid snowmelt in 2019

BC has experienced an average 1.4 °C warming from 1900 to 2013, with most of this warming attributed to the winter season (http://www.env.gov.bc.ca/soe/indicators/climate-change/temp.html). This is evident in ClimateBC predictions for the north face of Joffre Peak at 2400 m asl that show a positive trend in decadal mean annual temperature, with 0.3 °C increments starting at − 2.3 °C in 1970–1980 and continuing to − 0.6 °C in 2011–2018 (Fig. 6). By spring 2019, permafrost would have been almost completely degraded.

Local weather and snowpack data are available from October through May from the BC Ministry of Transportation and Infrastructure climate stations (https://pacificclimate.org) along Highway 99. With respect to longer term trends, we examined data from Blowdown Mid located at 1890 m asl on a NW aspect, 14.6 km to the northeast. The station has data from 1988 onwards, but useful hourly temperature and precipitation records start in 1994 and snow depth in 2003. The spring melt period from March 14 to May 13 was gap free and suitable for analysis. For melt seasons over the period of record, Table 1 shows temperature, precipitation, snow pack, and freeze thaw data. A freeze/thaw cycle was defined where the daily minimum and maximum temperatures were < − 2 °C and > 2 °C, respectively. Considering the record, there were no discernible trends; the 2019 spring snowpack, freeze-thaw days, and freeze-thaw range were not unusual, but the 2019 spring season temperature ranked 3rd warmest.

**Table 1:** Weather and snow, March 14–May 13 melt seasons, Blowdown Mid (1994–2019). For brevity, only last 3 years, plus summary statistics are shown

Year	Av. T (°C)	Precip. (mm)	Freeze/thaw days (#)	Daily range (°C)	Max snowpack (cm)	Max–May 13 snowpack (cm)
2019	0.91	77	32	11	167	70
2018	− 0.19	111	24	12	227	89
2017	− 1.55	214	36	11	271	60
Average	− 1.18	139	29	12	221	72
Minimum	− 4.24	28	17	10	150	32
Maximum	2.43	255	37	14	271	131

**Fig. 7:**
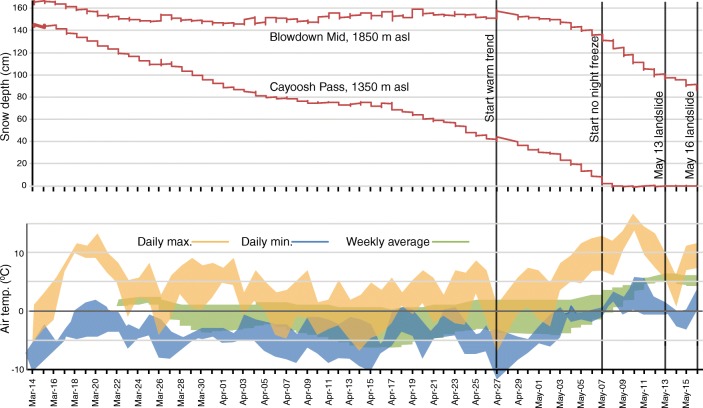
Daily temperature and snowpack data for the 2 months preceding the Joffre Peak landslides as recorded at stations within 15 km of the landslides and at elevations similar to those at the site of the failures. Colour envelopes enclose the elevation range 1350–2320 m asl. No snowpack data are available for the Blowdown Peak site. Data from MoTI: Cayoosh Pass, Station 26224, 1350 m asl; Blowdown Mid Station 26222, 1890 m asl; Blowdown Peak, Station 26221, 2320 m asl. Locations are shown on Fig. 1

Looking in more detail at spring 2019 (Fig. 7), weekly average temperatures at the elevation of the road (Cayoosh Pass; Fig. 1) were above zero, and the snowpack was in steady decline. At mid-elevation (Blowdown Mid; Fig. 1), a mid-March warm spell was followed in April by cool temperatures, with nighttime freezing and weekly average temperatures of 0 °C to − 2 °C. At the elevation of the peak (Blowdown Peak, 2320 m asl; Fig. 1), weekly average temperatures ranged from − 2 to − 5 °C. Throughout this time, the mid-elevation snowpack changed little, with ~ 150 cm snowpack depth. Beginning on 27 April, however, there was an abrupt warming; at all elevations, temperatures did not fall below zero after 4 May, the snowpack decreased rapidly, with valley-bottom snow gone by 8 May. At Blowdown Peak, temperatures remained above 0 °C for 7 days, from 7 am 7 May until 7 am 14 May, after which they briefly fell below 0 °C until 11 am 15 May, but there was no overnight freeze on 16 May.

In summary, permafrost was likely present on the north face of Joffre Peak, but decadal average temperature was in continuous decline since 1970–1980. By 2019 on the north aspect at the elevation of the failures, the decadal average temperature was approaching zero, and that melt season was the third warmest in the 31 years. Thus, permafrost degradation likely conditioned the slope to failure, with collapse on 13 May triggered by strong melt; melt may have also contributed to the 16 May collapse.

## Precursor activity

Photos provided by Ian Routley, of the Lillooet Natural History Society, from 1 May 2017 and 6 May 2019 (Fig. 2a, b), document a significant rockfall that occurred sometime between those dates from the face that would soon catastrophically fail. Review of Sentinel Hub imagery (https://apps.sentinel-hub.com) suggests this rockfall may have occurred between October 23 and November 7, 2018. On the afternoon of 13 May 2019 and the days thereafter, Brian Goldstone and Wayne Flann posted photos taken the previous week on the South Coast Touring Facebook page (https://www.facebook.com/groups/southcoasttouring/) that indicated areas downslope from the recent scar were being affected by rockfall which was noticeably dirtying the slope. This material was emanating from a wide vertical cleft on the left margin of the recent scar (Fig. 2b). The debris from this cleft source is visible on Sentinel Hub imagery taken in March 2019, but not earlier in the year.

## The 13 May landslide

**Fig. 8:**
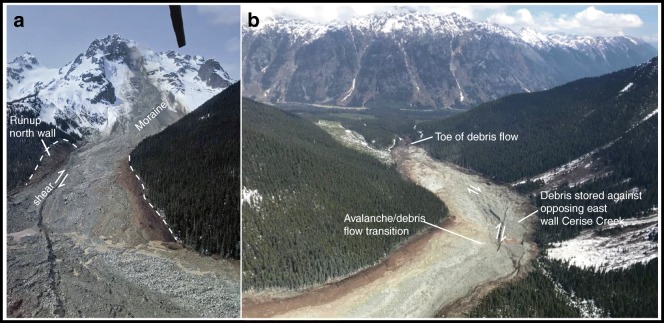
Photographs of the 13 May landslide posted on social media. **a** Photo by Wayne Pattern posted to South Coast Touring on 14 May, showing the run-up on the north valley wall from the very rapid frontal pulse and the shear developed between the debris that came to rest on the east side of Cerise Creek and the trailing flow. **b** Photo posted by Ken Nickel to Outside Magazine Instagram on 13 May providing a downvalley view of the deposit with the shear and the deposit on the opposing east valley side highlighted

The 13 May landslide was first noted by pilot Ken Nickel of Blackcomb Helicopters. His photos were posted to the Outside Magazine and South Coast Touring websites on the afternoon of the 13th (Fig. 8). Prior to the failure, the north face was bisected by a series of steep gullies with intervening rock buttresses (Fig. 2a, b). The buttress 230 m west of the summit slid away on a near-vertical foliation surface, leaving an approximately 130-m-wide by 180-m-tall scar (Fig. 2c). Analyses of seismic signals show that the failure happened at about 7:40 am local time on 13 May.

The photos suggest that the initial rockslide rapidly transformed into a careening flow that first overwhelmed the Little Ice Age moraine (Fig. 8a), super-elevated on the south-facing valley wall (Fig. 3), then flowed back up the north-facing valley side (Fig. 8a), and finally traveled down Cerise Creek to the distal deposition zone 4 km from the headscarp (Figs. 3 and 8b). The main debris lobe crossed Cerise Creek and came to rest on the east side of the valley (Fig. 8b), while the tail of the flow, which was confined to the west side of the valley, carried on, forming a right lateral shear as the debris streamed around a corner and down Cerise Creek (Fig. 8b). While most material stopped 200 m upstream from the forestry road that crosses Cerise Creek at 1320 m asl (Fig. 3), a portion continued to flow down the creek and became confined in a canyon before flowing over the alluvial fan at the confluence of Cerise and Cayoosh Creeks. Apart from a low-level aerial reconnaissance, no observations were made of the extent of the damage on the fan resulting from this first event.

## The 16 May landslide

**Fig. 9:**
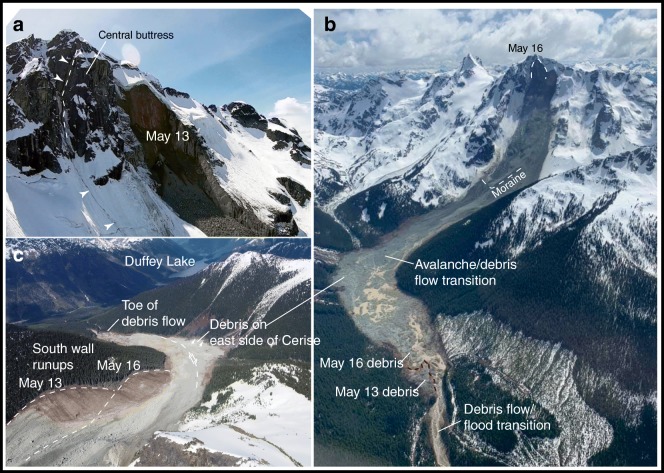
The 16 May Joffre Peak landslide. **a** The gaping vertical crack behind the central buttress, indicated by white arrows. The May 13 scar is ~ 130 m wide. Photo by David Safarik. **b** Photo posted by Phillip Binnema on 18 May showing the 16 May avalanche and debris flow track. From the top of the scar to the toe of the 16 May debris is ~ 4 km. The red line demarcates a remnant of the 13 May deposit. **c** The lower path showing adjacent 13 and 16 May run-up trimlines and material deposited on the east side of Cerise Creek. From the foreground to the toe of debris is ~ 2.7 km. Photo by Tom Millard

On 15 May, we flew over the 13 May landslide scar to assess ongoing hazards. In flight, we noted a gaping vertical crack behind and in line with the backscarp of the 13 May landslide on the east side of the central buttress (Fig. 9a) and several cracks that had formed in the summit snowfield on the west side of the central buttress. David Safarik posted a video shot taken from a fixed-wing aircraft on 15 May to Vimeo (https://vimeo.com/336646616) on 16 May. At 2:41 min elapsed time, a puff of dust is seen emanating from the gaping crack and there were dirt streaks in the snow below (Fig. 9a). These were signs of imminent collapse: the second landslide (Fig. 9b) happened the next day at 9:03 am on 16 May.

The source of the second landslide was approximately 150 m east of the first. The scarp of the 16 May event had a maximum width of approximately 180 m and a height of up to 190 m. The two failures combined created a planar backscarp up to 300 m wide. The 16 May rockslide struck the opposing valley wall just east of the location of the 13 May run-up zone (Fig. 9c). It then deflected to the east and ran more directly into the opposing valley side of Cerise Creek. However, as with the 13 May event, the trailing flow moved along a strike-slip fault as it bypassed the debris inside the corner (Fig. 9c).

Comparison of photos suggests that the second landslide stopped several tens of meters short of the limit of the 13 May event (Fig. 9b), but as in the case of the earlier event, some debris continued down into the canyon reach and onto the fan below.

## Continuing ice and rockfall activity

**Fig. 10:**
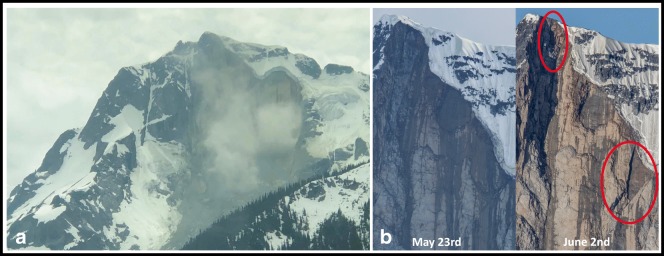
Photos posted to South Coast Touring. Rock and ice fall remain a hazard on and below the north face of Joffre Peak. **a** Rockfall at 2:51 pm on 2 June captured by Bob van Beers. The combined scar is about 310 m wide. **b** Photos by Steve Jones comparing the May 16 failure scar on 23 May (left) and after a rockfall on the morning of 2 June (right)

During the 27 May field visit, we noted rockfall at hourly intervals throughout the day. Posts on South Coast Touring on 24 May and 2 June document ongoing icefall and rockfall from the headscarp (Fig. 10a). A moderately large event modified the headscarp on 2 June (Fig. 10b). Rock and icefall continued through the summer of 2019 (see 6 Sept post to Instagram by Jim Sandford: https://www.instagram.com/p/B2F1Ci9j-bJ/), and remain a significant hazard affecting the face and immediate footslope.

## Landslide deposits in middle Cerise Creek valley

**Fig. 11:**
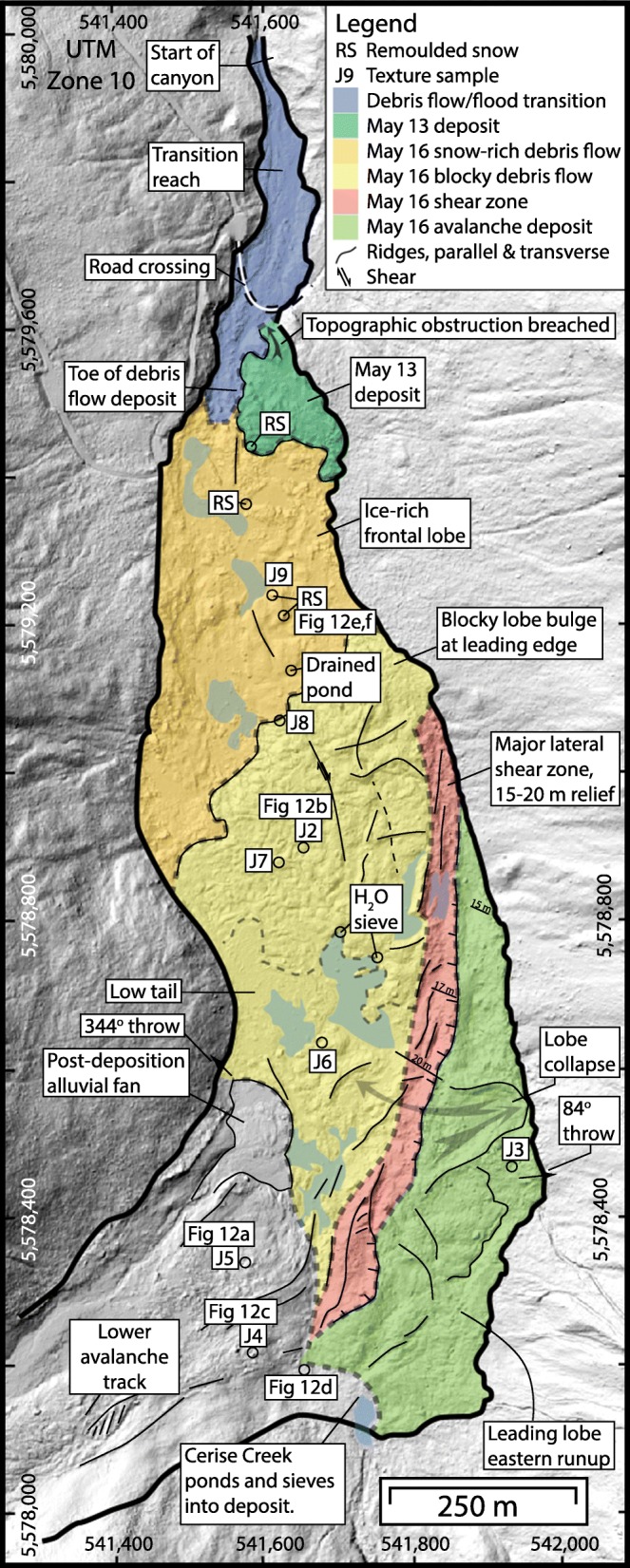
Geomorphic map of the May 2019 Joffre Peak debris flow deposit along middle Cerise Creek valley. Figure 13 gives results of texture analyses

The landslide deposits in middle Cerise Creek valley (Fig. 11) have an undulating to ridged morphology with a prominent shear escarpment 10–20 m tall, with the higher bench to the east formed by the avalanche phase that ran into and arrested against the west-facing opposing wall of middle Cerise Creek. The lower part to the west is underlain by debris that continued to flow as part of the debris flow phase. Total debris thickness ranges from 10–30 m along the formerly deepest part of the valley area affected.

**Fig. 12:**
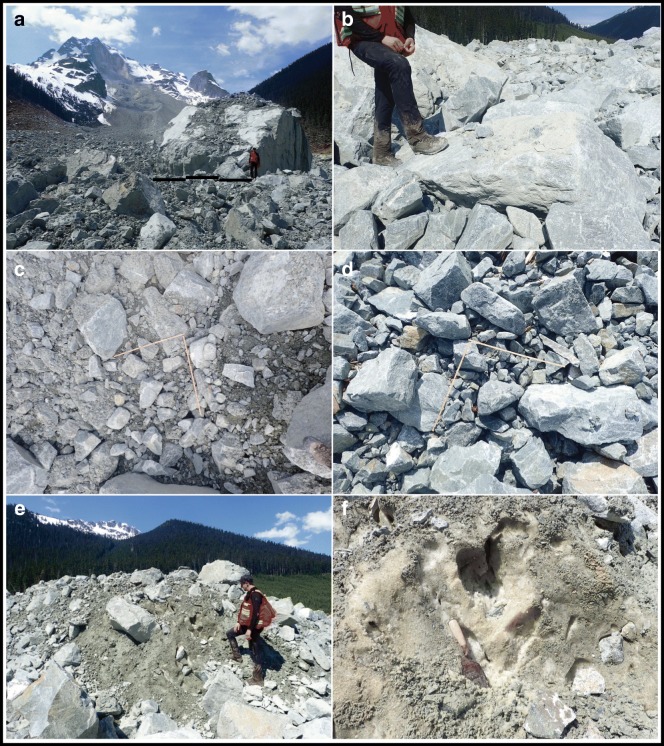
Photos of rock debris along middle Cerise Creek. **a** The largest blocks are megablocks up to 10 m across. **b** A gravelly muddy slurry-covered low surfaces, but in places, the mud was washed off (note line near boot), indicating a sloshing wet flow. **c** Typical deposit texture with muddy gravel filling interstices between blocks. **d** Less common open-work blocky debris. Ruler with perpendicular 1 m lengths for scale. **e** Compression ridge composed of snow mixed with rock debris in the lower 500 m of the 16 May landslide deposit. **f** Close-up view of Fig. 12e, showing the high proportion of snow relative to rock debris. See Fig. 11 for locations of sites. All photos by Pierre Friele

**Fig. 13:**
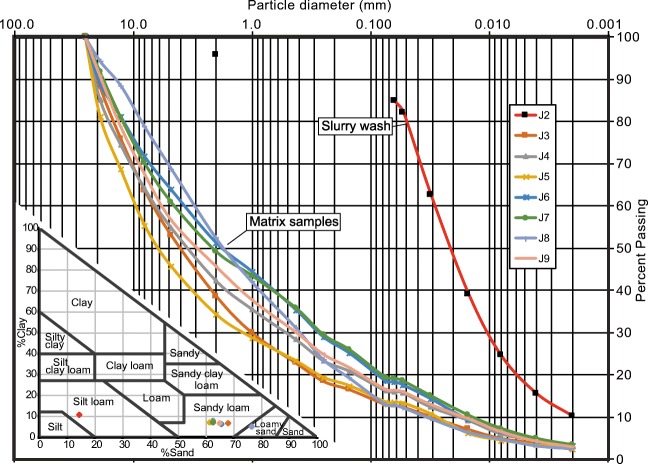
Grain size distribution of bulk matrix samples (< 25 mm fraction; see Fig. 11 for locations) and slurry wash drape (Fig. 12b). Inset texture triangle shows percentages of sand, silt, and clay in the samples. The bulk samples are ~ 45–65% gravel and ~ 3% clay, whereas the truncated samples (< 2 mm) are ~ 6% clay and classified as sandy loam

The debris comprises angular rock fragments ranging in size from clay to megablocks up to 10 m across (Fig. 12a). Most of the blocks have fresh angular surfaces, but some have oxidized facets indicating prefailure weathering surfaces. Most of the debris is coarse and matrix- to clast-supported with an interstitial matrix of gravelly sandy loam (Fig. 12c), but there are localized patches of open-work rock debris (Fig. 12d). Grain size analysis of the finer than 25 mm fraction indicates a matrix texture of 45–65% gravel, 20–40% sand, 10–15% silt, and 3% clay (Fig. 13). Samples truncated at 2 mm contain 6% clay, and the matrix would be classified as clay-rich, after Vallance and Scott (1997) who use a 5% clay proportion of the < 2 mm fraction to differentiate cohesive and noncohesive debris flows.

The 13 May landslide incorporated glacier ice and overrode and likely entrained a snowpack of 50–100 mm water equivalent over the entire 4 km length of its path. The 16 May landslide incorporated ice and snow over the upper 1500 m of its path before overrunning the earlier deposit; the distal 500 m of the debris deposit was rich in remoulded snow and rock debris that had been pushed to the front of the debris (Figs. 11 and 12e, f). Incorporation of ice and snow provided considerable free water. A ~ 10-mm-thick drape of mud covers low surfaces, but was washed from others (Fig. 12b), indicating a sloshing wet flow.

Visual observation at the outlet of ponds on the debris surface (Fig. 11) indicates water passes easily through the debris mass, indicating high porosity and permeability in these areas. The morphology of the deposit will change with time as the considerable interstitial snow melts, and surface and subsurface drainage patterns will change accordingly.

## Debris flow to debris flood transition reach

**Fig. 14:**
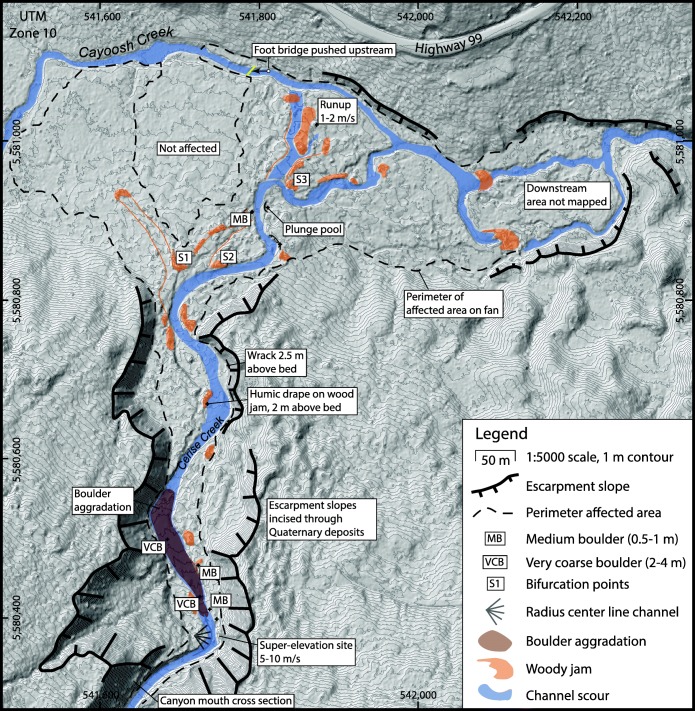
Cerise Creek alluvial fan showing damage corridor and site-specific observations mentioned in text. The affected area outline was covered by a blanket/veneer of humic debris, while the channel was scoured by timber jams forced by the flow

The downstream margin of the debris flow deposits (Fig. 11) forms a valley fill lobe 10–15 m thick. From this point, downstream 500 m is a transition reach feeding into a bedrock canyon (Figs. 3, 11, and 14). From 200 m wide at the toe of the debris, the path narrows to 100 m wide 200 m downstream at the destroyed logging road crossing, and 25 m wide 500 m downstream at the canyon head. Whereas the main debris flow deposit is a fill 10–30 m thick, in the transition reach, the channel was scoured with lateral levees of humic debris and timber. There are no thick bouldery diamict deposits or levees indicating passage of a pulverized rock debris flow. Rather, the area was affected by a slurry of mixed soil humus, ground wood, timber, and water, with a lesser amount of rock debris. This material was likely the leading edge of the debris flow, and simply carried on once the debris flow lobe arrested.

The canyon reach is 500 m long, 30–50 m deep, and is incised in Quaternary sediments (Fig. 14). The lower walls forming a rock-bound box canyon 5–10 m high and 20–30 m wide, with an average slope of 8°. Trimlines at the mouth of the canyon (Fig. 14) indicate that the flow exiting there was about 17 m wide, 4 m deep, and occupied a trapezoidal cross-section with an area of 60 m^2^.

## Deposits and features on Cerise Creek fan

The creek is incised in Quaternary sediments for another 550 m below the mouth of the canyon, but the valley floor gradually widens to 50–75 m and has an average gradient of 4.3°. For the purpose of the discussion, this narrow but unconfined alluvial reach is referred to as the upper fan; downstream where the fan properly radiates onto the Cayoosh Creek floodplain is referred to as the lower fan. The debris flood affecting the Cerise Creek fan covered an area of 13 ha and consisted of a plug of timber debris driven forward by a humic-rich slurry of reworked soil and comminuted woody debris.

About 100 m downstream of the mouth of the canyon, the flow super-elevated around a sharp bend (Fig. 14). A field survey of the run-up provides a velocity estimate based on the forced vortex equation (Chow 1959). With a run-up angle of 20° and a radius-to-channel centerline of 21 m, the equation for clear-water flow yields a velocity of 8.5 m/s. The equation generally applied to debris flows (Prochaska et al. 2008) yielded the same value if the *k* factor is held at 1. With higher *k* factors (2.5–5), the velocity estimate is 4–5 m/s. Applying the cross-sectional area of 60 m^2^ to velocities of 5–10 m/s gives a peak discharge at this point of 300–600 m^3^/s.

Using the regression of peak discharge vs. debris volume for bouldery debris flow (Bovis and Jakob 1999), we estimate a debris volume on the fan of 20,000–35,000 m^3^. With a deposit area of 13 ha and an average 0.5 m deposit thickness, then 65,000 m^3^ is estimated. While imprecise, the volume affecting the fan is on the order of 10^4^ m^3^, or 2 orders of magnitude smaller than the debris flow phase.

**Fig. 15:**
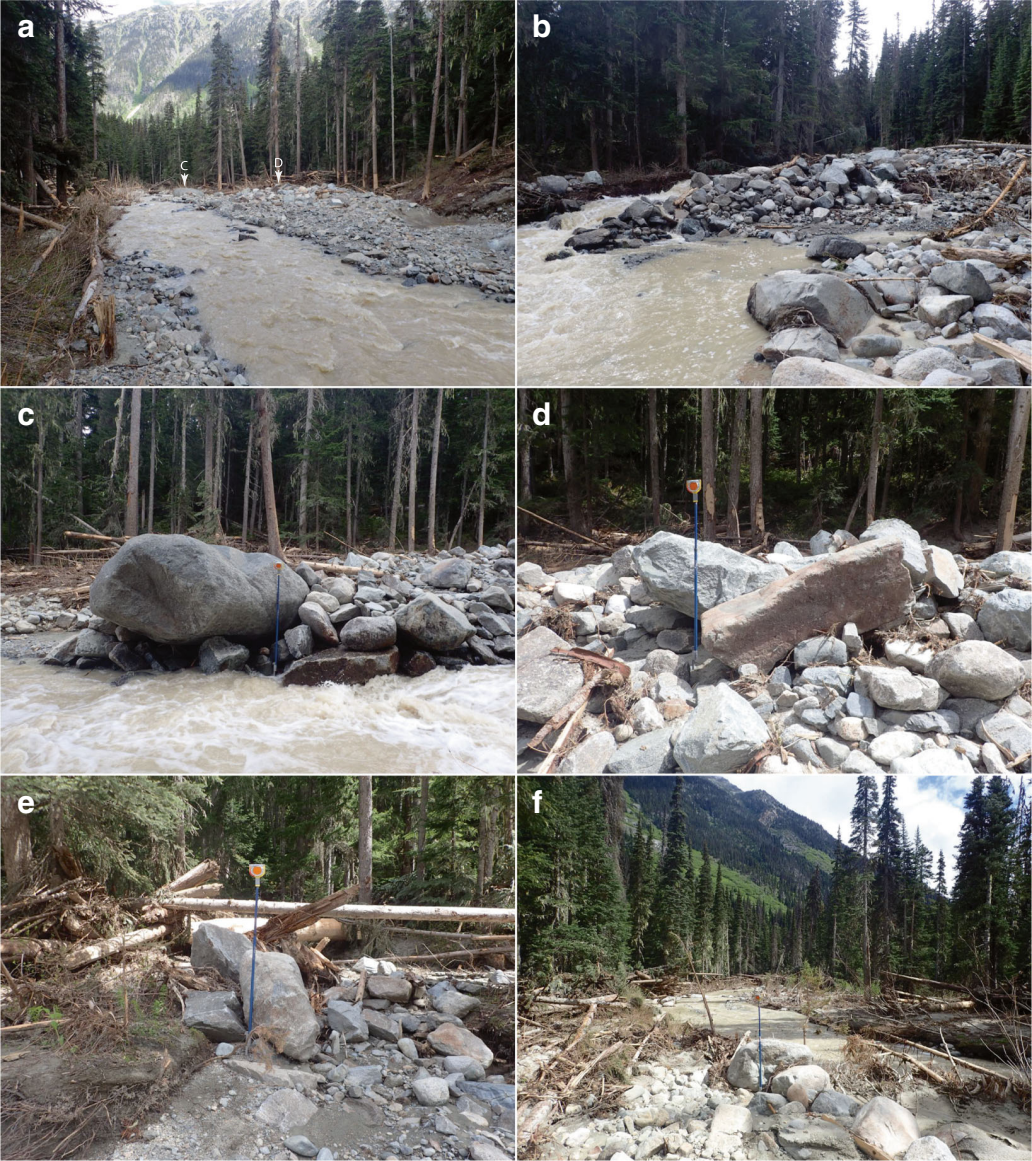
Cerise Creek fan apex. **a** View 100 m downstream from the super-elevation bend. Note the boulder accumulation at the right. Sites of examples shown in (c) and (d) are indicated. **b** View upstream at the distal edge of the boulder aggradation zone. Note erosive stripping of trees adjacent to the channel. **c** Large (3 m) amphibolite boulder. **d** 0.75 m block aligned parallel to flow. **e** Imbricate subangular blocks up to 0.75 m across. **f** Boulder train on the stripped surface of the lower fan; boulders are up to 0.75 m across. Staff in photos c–f is 1.5 m high. All photos by Pierre Friele

Over the 150 m length of the reach below the super-elevation bend, the flow widened to 30 m within the 50–75 m width of the valley floor (Fig. 14). A boulder gravel sheet, lacking lateral debris levees, filled the channel in this reach, and suggests that the flow at that point was a debris flood (Fig. 15a, b). Many boulder clusters contained imbricated clasts up to 0.75 m across (Fig. 15c–f). At two locations, large boulders had medial diameters of 3 m (Fig. 15c). Applying Costa’s (1983) paleoflood power function to estimate velocity from boulder size gives velocities of 4–5 m/s for the largest imbricated clasts and 9 m/s for the two largest boulders. These values are in agreement with the run-up estimates of 4.5–8.5 m/s.

**Fig. 16:**
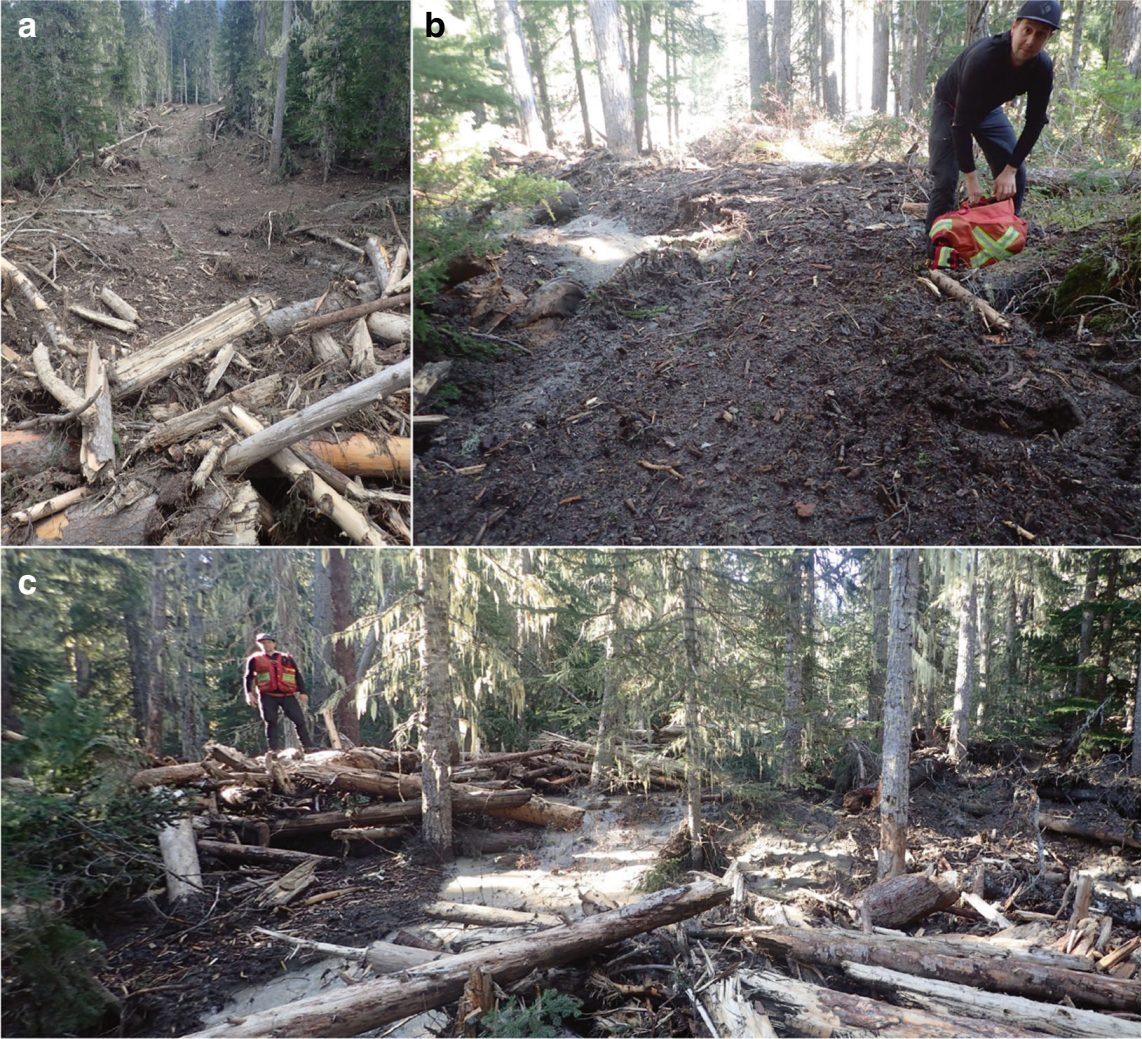
Damage on the Cerise Creek fan. **a** View upstream of 10-m-wide swath in forest cut by a flow laden with woody debris. **b** Typical deposit, ca. 50 cm thick, of humic debris consisting of reworked soil and comminuted wood. **c** Coarse wood debris with a humic slurry veneer and a thin sandy wash. All photos by Pierre Friele

The main plug followed the original stream channel on the fan, pushing woody jams out of the channel at bends. At one point on the central part of the fan, a large jam split the flow (see label S1, Fig. 14). The main pulse continued northeast down the channel, but a smaller surge moved northwest out of the channel, creating a swath 10 m wide and 100 m long through forest before coming to rest (Fig. 16a). The main front following the original channel bisected at jams at two more locations downstream (see labels S2 and S3, Fig. 14). The swath-cutting plugs all stopped within meters of Cayoosh Creek. The fan surface along the thalweg of the main flow is heavily scoured, with dry plunge pools, eroded banks, bark-stripped trees 1–2 m above ground level, and out-of-channel imbricate boulders up to 0.75 m across (Fig. 14).

In distal overbank areas (Fig. 16), a few swaths were created by woody debris (Fig. 16a), but most of the fan area was washed by a humic-rich debris flood 0.5–2 m thick that left no significant amount of mineral sediment apart from localized, thin sand deposits (Fig. 16b, c). Velocity head from run-up on trees suggests velocities of 1–2 m/s for this humic flow. The flow entered Cayoosh Creek over a 500-m channel length, inducing a surge with trimlines up to 1 m high on the floodplain. The Cerise Creek trail footbridge, which crossed Cayoosh Creek midway along the fan margin, was pushed 25 m upstream (Fig. 14). At the downstream fan margin, a large woody jam formed on Cayoosh Creek, forcing a partial avulsion down a side channel (Fig. 14). No significant damage was noted at the highway bridge 3 km downstream, apart from silt lines that indicate a bankfull surge. No woody debris or gravel was carried into Duffey Lake, but the water became turbid with fine sediment.

## Volume estimates

We estimated volumes for the two events from pre- and post-event photos and Lidar topographical models for the headscarp area above 2050 m asl. Using Agisoft Metashape (Agisoft 2019), we developed photogrammetry models for the pre-event topography from three sets of photos: government air photos taken in 2017 and handheld camera photos taken from helicopter after the first event on 15 May 2019 and after the second event on 18 May 2019. We aligned the pre-slide topography to the Canadian digital elevation model (CDEM), and the 15 May and 18 May models to the pre-slide model, using CloudCompare software (CloudCompare 2019). The root mean squared (RMS) error for the alignment was 5.65 m, 1.94 m, and 0.90 m, for the pre-event, to CDEM, 15 May to pre-event, and 18 May to pre-event, respectively. The volume difference for the 13 May and 16 May events was also calculated using CloudCompare which are, respectively, 1.9 × 10^6^ m^3^ and 3.1 × 10^6^ m^3^. The 2σ error estimates for the first event comes to 0.43 × 10^6^ m^3^ and the second event was 0.46 × 10^6^ m^3^, or 23% and 15%, respectively.

We made an independent estimate of the total volume of the two landslides using aerial Lidar data collected on 22 February 2018 and 26 May 2019. The total rock mass lost at the source is 5.2 × 10^6^ m^3^, and the accumulated material in the deposit is 4.9 × 10^6^ m^3^ with respective uncertainties of ± 2.01% and ± 13.65%. The source and deposit uncertainties reflect the standard deviation (± 1σ) of stable terrain following methods described elsewhere (Menounos et al. 2019). While there is good agreement between the total source volume estimates from the photogrammetry and Lidar analyses, the deposit volume is smaller than expected considering that bulking and dilation would have occurred. Expressing errors at 2σ gives 27% for the deposit, suggesting that there is insufficient precision in the deposit estimate to account for bulking and dilation.

## Travel angles

The travel angles for the two events are similar: about 0.28 from the headscarp to the toe of the blocky debris flow deposits in middle Cerise Creek and 0.22 to the limit of debris flood deposits at the distal margin of the Cerise Creek fan. The reach matches the mean for large debris avalanche/debris flow events in the Canadian Cordillera reported by Geertsema and Cruden (2014), but is shorter than debris flow mean (H/L = 0.21) compiled by Corominas (1996). The additional reach of the debris flood phase is noteworthy as it extended the hazardous zone to the fan. Joffre was indeed a rare and dangerous event (Jackson et al. 1987; Jakob et al. 2016). Process chain reactions, such as the flood surge on Cayoosh Creek, further extended the reach.

## Seismic analysis

**Fig. 17:**
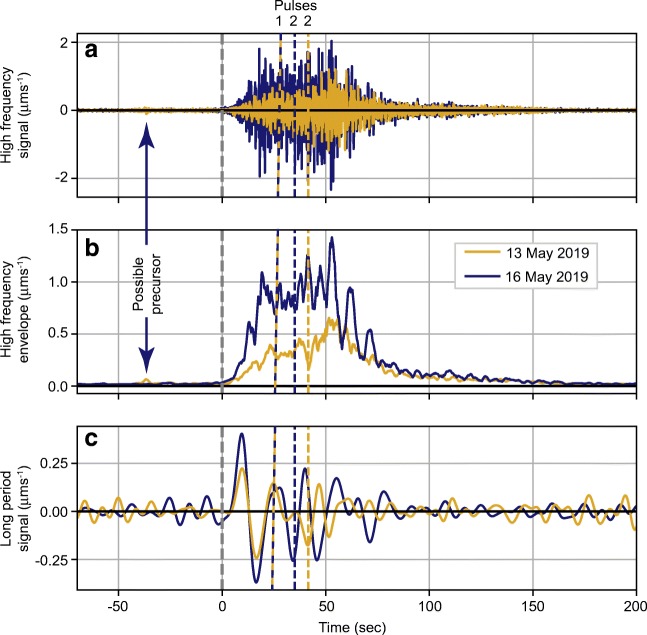
Comparison of the seismic signals of the two Joffre Peak landslides as recorded on the vertical component (HHZ) of LLLB, a Canadian National Seismograph Network (CN) broadband seismic station located about 50 km northeast of the landslide source (see Fig. 1). **a** 1–10 Hz bandpass-filtered seismic signals. **b** Smoothed envelope of the 1–10 Hz signals shown in panel **a**. **c** Long-period (0.01–0.1 Hz) signals. All traces have been corrected for station response to physical units. Zero time corresponds to 14:40:06 UTC (7:40:06 am local) for the 13 May event and 16:03:12 UTC (9:03:12 am local) for the 16 May event. Dashed yellow and purple lines indicate the approximate separation between the two pulses described in the text for the 13 May event and 16 May event, respectively

Seismic signals generated by these events were detected up to hundreds of kilometers from the source. The locations of the two closest seismic stations, WSLR and LLLB (broadband stations that are part of the Canadian National Seismograph Network (CN)), are shown in Fig. 1. Although WSLR is closer to Mount Joffre, at about 41 km, the signals from LLLB, about 50 km from the source, are clearer and slightly higher in amplitude due to the latter station’s lower noise levels, and also possibly because of lower attenuation in that direction, and because the rock avalanches moved towards LLLB (to the NE) and away from WSLR (SW). We focus here on the seismic signals from LLLB (Fig. 17).

As is typical for landslide seismic signals, the waveforms for both Joffre Peak events have emergent first arrivals, making it hard to accurately pick seismic wave arrival times. The exact time that the signal emerges from the noise depends on the background noise level of the seismic station. Because LLLB had lower noise levels, the signals emerged earlier at that station than at WSLR, specifically at 14:40:06.0 UTC (07:40:06 PDT) for the 13 May event and 16:03:12.0 UTC (09:03:12 PDT) for the 16 May event. These times correspond to the zero time on the time axis of Fig. 17. The true start times of the events are actually earlier by up to ~ 30 s or more because of the combined effect of the emergent onset and seismic wave travel times, which would be ~ 15–25 s for surface waves traveling at ~ 2–3 km/s. The 13 May event was preceded by a faint signal starting ~ 40 s prior to the onset of the main event, suggesting a smaller collapse immediately before the main event.

The seismic amplitudes of the 16 May event were higher than those of the 13 May event, indicating that it was larger and/or more energetic. Yet the total durations of the energetic parts of the two events were likely very similar: the high-frequency signals of the two main events are similar in duration, both fading back into the noise after about 160 s. The duration of the high-frequency signal can be a good approximation of the duration of rapid movement for simple events (e.g., Hibert et al. 2011), although the lack of near-source stations means that additional movement that was not energetic enough to be observable tens of kilometers from the source may have continued after ~ 160 s. Debris flows tend to be much quieter seismically than rock avalanches for a given volume (Allstadt et al. 2018); thus, the later, more flow-like parts of these landslides may not have generated observable seismic signals.

The shape of the envelope of the signals of the two events consists of two pulses of energy (Fig. 17a). The first pulse peaked after about 24 s in both cases, then flattened or decreased slightly until the signal started rising again. The time of the second pulse was delayed for the 13 May event relative to the 16 May event. For the 13 May event, the high-frequency signal began to rise again at around 42 s, peaking at about 52–56 s. In comparison, for the 16 May event, the signal began to rise again at ~ 35 s, peaking broadly between ~ 35 and 55 s. The second peak of the 13 May event had a higher amplitude than the first peak, while the second peak for the 16 May event was less distinct and closer in amplitude to the first peak. Although two pulses of energy can sometimes indicate two distinct, but closely timed collapses, the consistency of this pattern between the two events and the lack of field evidence for a multi-part collapse for either event indicate that the first pulse corresponds to the build-up of momentum after initial mobilization and the second pulse corresponds to material running up against the south-facing valley wall at high velocities.

Additional information about timing and interpretation comes from the long-period (> 10 s, < 0.1 Hz) signals (Fig. 17c). Clear long-period signals are generally observable only for rapid landslides with volumes larger than 10^5^–10^6^ m^3^ (Allstadt et al. 2018) and are the result of forces exerted on the Earth by large-scale, coherent bulk accelerations of the flowing material as it initiates, decelerates, and makes turns and interacts with large-scale features of terrain at high velocities. The forces are proportional to the total mass and the bulk acceleration (Kawakatsu 1989). Comparison of the long-period signals of the two events (Fig. 17c) shows that the two signals are remarkably similar in shape for the first ~ 20 s, but the 13 May signal is lower in amplitude. This implies that the two events shared a similar initial mechanism, trajectory, and duration of mobilization, but the 13 May event had a smaller mass and/or a lower initial acceleration rate. After this point, the long-period signals diverge, likely reflecting the different run-up locations and slightly different trajectories the two landslides followed. The long-period signal lasts slightly longer for the 16 May event, ~ 80 s compared with ~ 65 s for the 13 May event, although the difference could be related to the lower signal-to-noise ratio of the 13 May event. In any case, the relatively short long-period signal durations relative to the 160-s duration of the high-frequency energy suggest that large-scale bulk accelerations were over within just over a minute, even though continued smaller-scale energetic motion continued for another ~ 80–100 s.

Given what we know about the sliding paths (Figs. 2 and 10), the end of large-scale bulk motions likely occurred around the point where the deposits ran up against the opposing east wall of Cerise Creek (Fig. 11), ~ 3 km from the source area for both events, including super-elevations. Given the duration of long-period motion of ~ 65 s and ~ 80 s for the 13 May and 16 May events, respectively, we obtain rough average velocity estimates of ~ 46 m/s and ~ 38 m/s from source area to the opposing wall of Cerise Creek. Based on the characteristics of the deposits and damage to trees, motion that was still energetic enough to have generated the observable high-frequency seismic signals that lasted for ~ 160 s likely continued until the main debris flow lobes stopped 200 m upstream of the logging road. Tracking along the center of the estimated flow path yields travel distances of ~ 4320 m for the May 13 event and ~ 4000 m for the May 16 event (Fig. 3), and average overall velocities of ~ 27 m/s and ~ 25 m/s, respectively.

## Possible contributing factors

### Climate change and alpine permafrost degradation

Surface characteristics such as snow cover and slope aspect play a strong role in permafrost presence (Noetzli and Gruber 2009; Hasler et al. 2015), and models (Gruber 2012a, b; Hasler and Geertsema 2013) suggest it was likely present on the north face of Joffre Peak. Since 1970–1980, decadal average temperatures have risen incrementally, contributing to permafrost degradation. By 2019, permafrost in the landslide headscarp was likely severely degraded, and close to zero (Fig. 6). Despite these indications, we cannot prove that permafrost was present; however, associations between permafrost degradation and increased landsliding have been suggested, and sometimes verified, for other sites in BC (Geertsema et al. 2006; Cloutier et al. 2017), Alaska (Coe et al. 2018), and Europe (Gruber and Haeberli 2007; Huggel et al. 2012). Therefore, permafrost degradation was likely a conditioning factor.

### Weather

The 2019 snowmelt season was the 3rd warmest in 31 years; however, there was no discernible trend over the period of record. With the above average melt, water may have gotten deeper in the rock mass than during previous spring seasons and increased the likelihood of impacting any residual permafrost.

The first landslide happened 17 days after the onset of a warming trend and 9 days after nighttime freezing ceased at mid and high elevations. Snowmelt likely elevated pore pressures in the joint systems that formed the failure plane of the 13 May event. Meltwater would further degrade mountain permafrost. Although temperatures fell slightly between the 13 and 16 May failures, weekly average temperatures were still 5–7 °C and the snowpack continued to melt. Interestingly, both landslides happened in the morning hours, perhaps reflecting a lag in the diurnal snowmelt pulse. Thus, snowmelt and high pore pressures may have contributed to the 16 May landslide as well.

### Debuttressing from retreat of alpine glaciers

The alpine glacier on the north side of Joffre Peak has thinned and retreated, debuttressing the lower part of the slope that failed. Over a longer period, that glacier has also eroded and significantly steepened the slope, which may have contributed to progressive failure mechanisms, whereby fractures and joints within the rock mass grow and coalesce over time, weakening the rock mass (Eberhardt et al. 2004; Holm et al. 2004).

### Debuttressing from progressive failure

A small but significant collapse occurred sometime between May 2017 and 2019 (Figs. 2a, b). The 13 May 2019 collapse reduced structural support for the central buttress (Fig. 2). During the helicopter overview flight between the two events, we noticed a gaping fracture on the east side of the buttress (Fig. 9a) as well as a series of cracks in the summit glacier and snowfield on the west side of the buttress. Thus, the 16 May landslide was probably forced by both snowmelt and debuttressing due to the 13 May landslide.

## Impacts

The landslide occurred within a nature conservancy with high recreation values. The collapse destroyed the north face of Joffre Peak, which hosted at least three popular alpine couloir climbing and extreme ski descent routes (https://gripped.com/news/massive-feature-on-joffre-peak-in-b-c-collapsed/). Further, about 75 ha of forest was destroyed by the debris flow, representing a significant loss of wildlife habitat and snow avalanche protection forest for backcountry skiers transiting middle Cerise Creek to Keith’s Hut in upper Cerise Creek (Fig. 3). There were separate summer and winter trails through the middle reach of Cerise Creek to Keith’s Hut, with each trail losing about 1 km length. The course of middle Cerise Creek was obliterated, and drainage reestablishment may lead to a lengthy turbidity pulse in lower Cerise Creek and Cayoosh Creek downstream to Duffey Lake.

Downstream of the canyon reach, debris floods affected ~ 13 ha of the Cerise Creek fan with 2 ha of channel and floodplain scour, abundant woody jams along channel margins, and an 11-ha area with a veneer (< 1 m) of humic organics and large woody debris in the understory. About 300 m of the access trail was destroyed on the fan, and a surge of water and wood entered Cayoosh Creek, pulsing upstream ~ 125 m and destroying the footbridge at the trailhead. Closure of affected trails has been recommended within the Cerise Creek Conservancy for 1 year, to be followed by a risk assessment by a geohazard specialist in 2020.

## Residual hazard

**Fig. 18:**
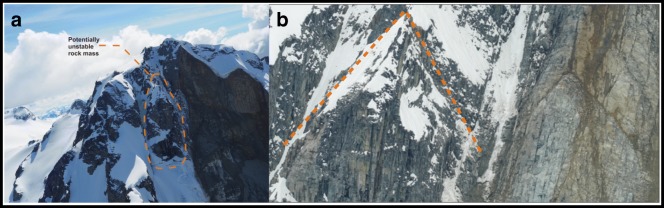
**a** Residual hazard identified by Drew Brayshaw in CBC News interview on 22 May 2019. Photo by Tom Millard. **b** Inverted V-shaped structural features on the right are mimicked by the triangular shape of the buttress on the left (highlighted by dashed line). Photo by Conny Amelunxen

A third potential failure site was identified by Professional Geoscientist Drew Brayshaw in a CBC News interview on 22 May 2019 (https://www.cbc.ca/news/canada/british-columbia/joffre-peak-landslide-imminent-1.5145409). He pointed out structural features on a third buttress east of the 16 May failure that suggested that the failure plane continued behind this feature (Fig. 18). Although this is true, it does not appear open and a third failure is judged not to be imminent.

The primary asset in this area is Highway 99. The fahrboshung angle from the Joffre Peak source area to the highway is 0.2 and the highway grade is 19 m above the distal margin of the Cerise Creek fan. Based on these values, a landslide of sufficient volume to reach Highway 99 is unlikely (Mitchell et al. 2019).

## Conclusions

Social media played a large role in rapidly alerting the geoscience community to the occurrence of the May 2019 Joffre Peak landslides, in southwest BC, allowing rapid photogrammetric data collection and hazard/risk assessment. Citizen science observations included photo and video documentation of precursor activity for both events, the first headscarp photos, initial tracings of the paths, a field report of trail damage on the fan, and ongoing documentation of post-event rockfalls.

Herein, we have presented an initial assessment of the events. Precursor rockfall activity started at least ~6 months prior to the 13 May 2019 landslide and was noticeably active in March and April 2019. The May landslides were spaced three days apart; each involved 2–3 × 10^6^ m^3^ of rock on the steep north slope of Joffre Peak. Although conditioned by factors such as unfavourable structure, rock mass weakening by chemical weathering, and alpine glacier erosion and retreat, the trigger appears to have been rapid snowmelt. The source area was sufficiently high in elevation and north-facing that alpine permafrost was likely present. Since 1970–1980, there has been decadal degradation of alpine permafrost, with ClimateBC predictions indicating mean annual temperature at 2400 m on the north face gradually increasing to from − 2.3 to − 0.6 °C. Although direct evidence is lacking, alpine permafrost degradation was likely a contributing factor, while the rapid snowmelt in 2019 may have tipped the balance.

Landslide nomenclature is categorical, but the process is a continuum. This leads to difficulties in process classification. Most rapid landslides are complex, with fall, slide, avalanche, and flow phases. It is the high (excess) mobility that distinguishes an avalanche. Here, we aggregate the initial fall/slide into the avalanche phase. The May 2019 Joffre landslides were similar, starting as rock avalanches, rapidly transforming to large debris flows, and concluding as distal organic-rich debris floods. Although separated by days, the two events might be viewed as one event, a two-part sequence with the second event dependent on the first because of debuttressing.

The upper track, 1500 m long from the headscarp at 2600 m asl to the Neoglacial moraine at 1650 m, has an average slope of 26°. The initial acceleration of the fall/slide was impeded by the Neoglacial moraine and then the opposing wall of the Joffre North tributary. The transition from a rock avalanche to a very large debris flow likely occurred along the tributary downstream of the Neoglacial moraine, where over this 1500-m-long reach, the average slope is 9°. At the confluence of the Joffre North tributary and the middle reach of Cerise Creek, at 1370 m asl is an abrupt concave break to 3° slope and a sharp northerly bend; this marks the terminus of the avalanche phase with deposition against the opposing east wall of Cerise Creek.

The debris stored along middle Cerise Creek extends over 43 ha, and is 1500 m length and 200–400 m in width. The avalanche which impinged directly on the opposing east valley side forms a higher bench 14 ha in area, spread 750 m along the opposing valley wall. The lower western and northern portions, covering 29 ha, represent the debris flow phase, with the 10–15-m-thick and 200-m-wide terminal lobe located about 200 m upstream from the logging road crossing. After debris flow deposition, the seismic signal attenuated, coincident with the transition to a debris flood composed primarily of humic debris, timber, and water. About 13 ha on the Cerise Creek fan were affected by this debris flood.

The travel angle for the avalanche/debris flow portion was 0.28, whereas the travel angle to the limit of the debris flood was 0.22. Although the debris flow matrix (< 2 mm fraction) was classified as cohesive (> 5% clay) and was saturated with excess sloshing muddy water, the travel was not unusual for catastrophic landslides. Path obstructions may have reduced travel.

The events were recorded by nearby seismic stations, with each trace lasting approximately 160 s to the end of the debris flow phase. The landslides may have reached 40–50 m/s velocity along their upper tracks, and averaged 25 m/s from headscarp to the distal margin of the debris flow deposit. The debris flood on the upper fan had an estimated velocity of 5–10 m/s diminishing to 1–2 m on the lower fan.

While there appears to be no imminent threat of another catastrophic (> 1 Mm^3^) Joffre Peak collapse, the north face continues to spawn debris, and this creates a hazard to recreation users. The Cerise Creek Conservancy will be subject to closure for 1 year, when the risk management plan will be reconsidered. The May 2019 Joffre Peak landslides may be one more of a growing list of events responding to human-induced climate change factors (glacier retreat, permafrost degradation, extreme weather), and indicates that we should expect more large landslides to occur throughout the Coast Mountains in this century.
